# Sellar and Parasellar Metastatic Tumors

**DOI:** 10.1155/2012/647256

**Published:** 2011-10-13

**Authors:** Tamer Altay, Khaled M. Krisht, William T. Couldwell

**Affiliations:** Department of Neurosurgery, University of Utah, 175 N. Medical Drive East, Salt Lake City, UT 84132, USA

## Abstract

The sellar and parasellar (SPS) region is a complex area rich in vital neurovascular structures and as such may be the location of first manifestation of a systemic malignancy. Metastases to this region are rare; breast cancer is the most common source among those that metastasize to the SPS region. Ophthalmoplegia, headache, retroorbital or facial pain, diabetes insipidus, and visual field defects are the most commonly reported symptoms. Lack of specific clinical and radiological features renders SPS metastases difficult to differentiate from the other frequently encountered lesions in this area, especially when there is no known history of a primary disease. Currently accepted management is multimodality therapy that includes biopsy and/or palliative surgical resection, radiation, and chemotherapy. Although no significant survival benefits have been shown by the surgical series, surgical resection may improve quality of life. Here we review the relevant literature and present six illustrative cases from our own institution.

## 1. Introduction

Metastatic lesions comprise approximately 1% of the tumors in the sellar/parasellar (SPS) area for which patients undergo transsphenoidal surgery (TSS) [1, 2]; however, it has been reported in autopsy series that the rate of metastasis to these areas could be as high as 28% [3]. Breast and lung cancer are the two most common types of malignant tumors that metastasize to the SPS region, with respective rates of 40% and 33% [4]. Metastases of prostate [5], renal cell [6], gastrointestinal [7], thyroid [8, 9], and pancreatic cancers [10], and lymphoma [11], leukemia [12], melanoma [13], and plasmocytoma [14] have also been reported.

Despite the advancement in the imaging modalities, tumors that have metastasized to the SPS areas may still be difficult to differentiate from pituitary adenoma on radiographic studies [2, 14, 15]. Thickening of the pituitary stalk and invasion of the cavernous sinus may be suggestive of such lesions, but invasion of the cavernous sinus may commonly occur with pituitary adenomas. This distinction is also clinically challenging, although there are very few symptoms that suggest a metastatic lesion. 

Management options are multimodal and vary depending on whether a primary source is known or on the likely differential diagnoses based on the clinical and radiological findings. Multimodal options include radiation therapy, chemotherapy, and/or surgery [16, 17], although the tumor invasiveness renders surgical resection limited. Although surgical series have not shown any survival benefits, the patient's quality of life may be improved [10, 14].

In this paper, we review the clinical, endocrine, and radiological features of the metastatic SPS tumors with currently accepted therapeutic options based on the pertinent literature. In addition, we report six cases from our institution and discuss their management with long-term clinical outcome.

## 2. Materials and Methods

A systematic review of the literature was performed using PubMed and the bibliographies of reviewed articles. The medical records of six patients admitted to the University of Utah Health Sciences Center between 2001 and 2011 were reviewed retrospectively. Clinical presentation, radiographic studies, treatment, histopathological confirmation, outcome, and prognosis were recorded (Table 1). The institutional Review Board approval was granted for this retrospective clinical paper.

## 3. Results

### 3.1. Patient 1

**Figure 1:**
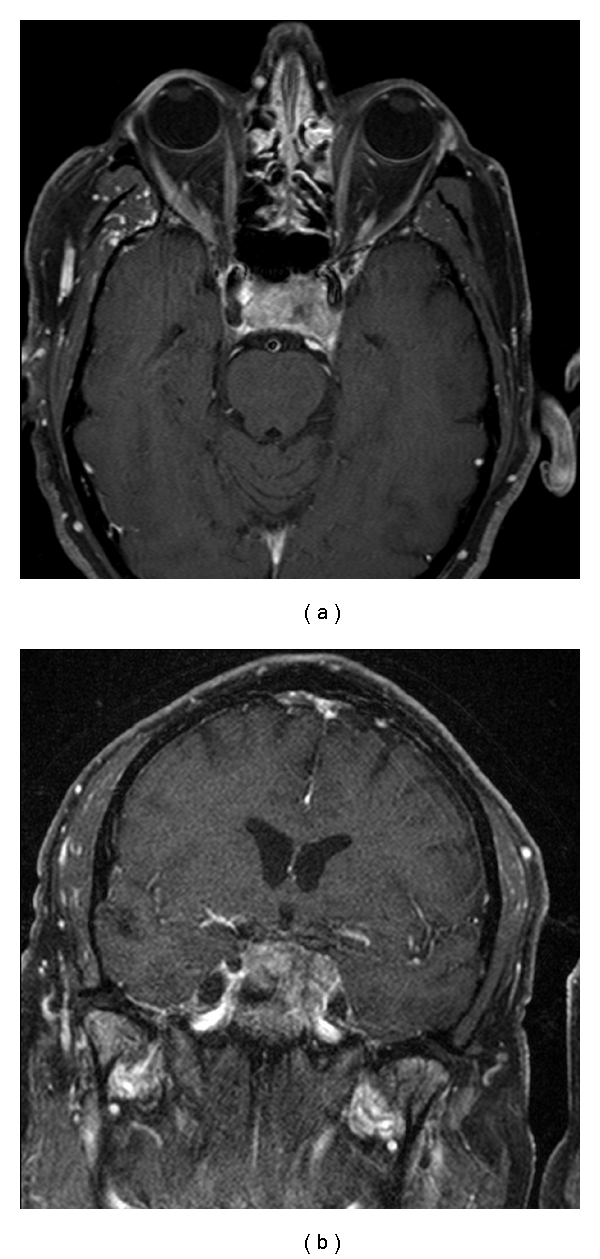
Axial and coronal T1-weighted MR imaging of the brain with gadolinium enhancement showing a heterogeneously enhancing mass involving the sella with invasion into the left cavernous sinus and the superior clivus.

A 77-year-old man with known prostate cancer presented with a four-month history of left retro-orbital pain followed by left eye ptosis. At presentation, he had complete left third nerve palsy. His visual acuity was intact in both eyes, with full visual fields to confrontation. Brain magnetic resonance (MR) imaging (Figure 1) showed a heterogeneously enhancing mass lesion that measured 29 × 17 × 29 mm involving the sella, with invasion of the left cavernous sinus and the upper clivus. The lesion extended to the inferior orbital fissure and was centered in the sella turcica and the cavernous sinus. A biopsy was obtained via a transnasal transsphenoidal approach. A diagnosis of metastatic prostate carcinoma was made. He subsequently underwent chemotherapy and focused radiation to the sellar region and was noted to have stable neurological examination findings two months after surgery, with no change in his ophthalmoplegia.

### 3.2. Patient 2

**Figure 2:**
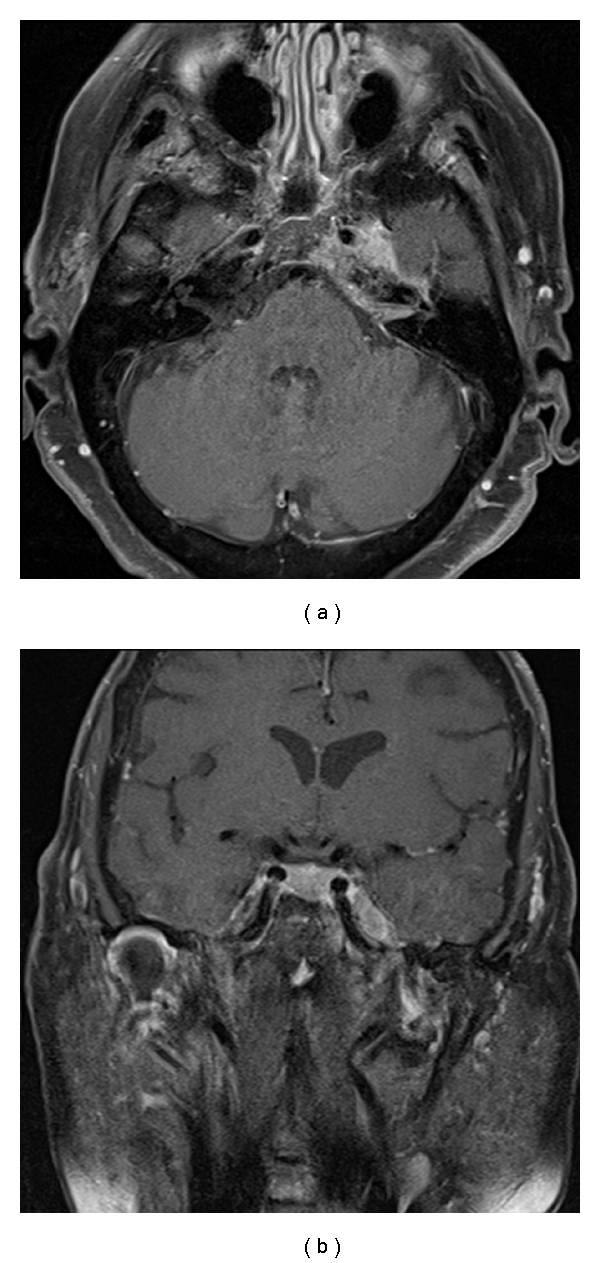
Axial and coronal T1-weighted MR imaging of the brain with gadolinium enhancement demonstrating a heterogeneously enhancing left petrous apex mass with extension into the adjacent middle cranial fossa and cerebellar pontine angle.

An 82-year-old woman with known history of breast cancer presented with several weeks' complaint of progressive left-sided hearing loss as well as facial pain and numbness in the first and the second divisions of the trigeminal nerve, respectively. On neurological examination, left-sided hearing loss and facial numbness along the V1 and V2 distributions were confirmed. MR imaging showed evidence of a heterogeneously enhancing mass in the left petrous apex that extended to involve Meckel's cave, the lateral cavernous sinus, and the internal auditory canal (Figure 2). A left frontotemporal craniotomy was performed for biopsy. The histopathological evaluation was consistent with a metastatic adenocarcinoma of the breast. She was discharged home on postoperative day three in stable condition for followup with oncology.

### 3.3. Patient 3

**Figure 3:**
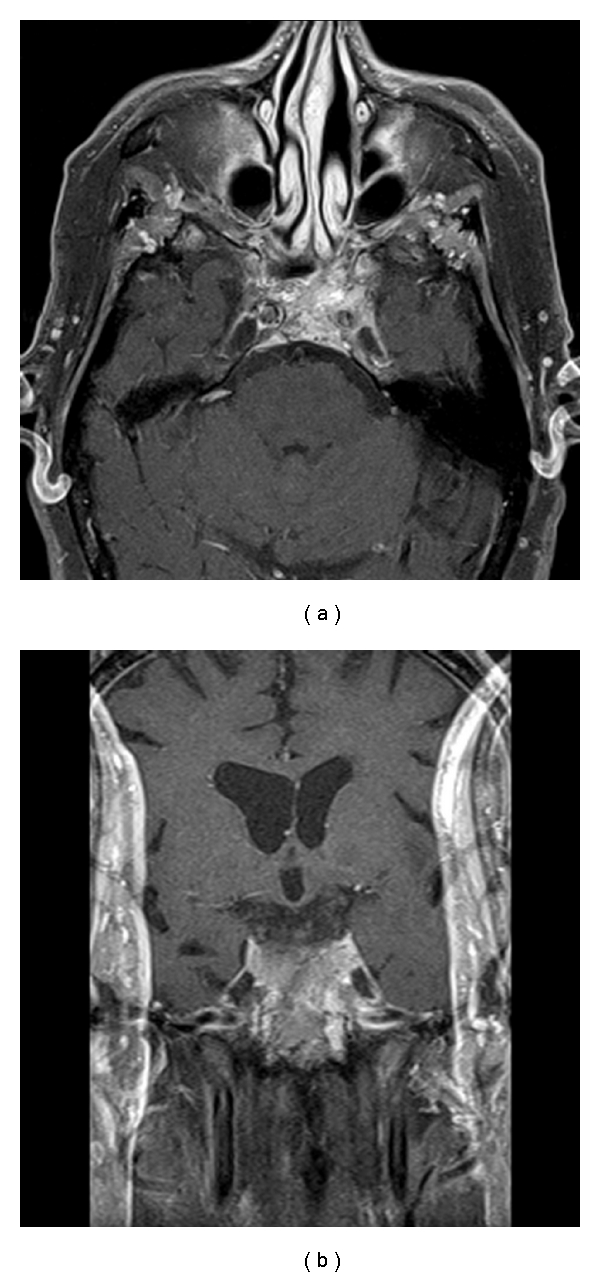
Axial and coronal T1-weighted MR imaging with gadolinium enhancement showing a homogenously enhancing soft tissue mass involving the clivus, pituitary fossa, cavernous sinus, and posterior nasal cavity.

A 79-year-old man with known history of prostate cancer presented with several weeks' history of progressively worsening double vision and eventual right eye ptosis. Neurological examination revealed complete third and sixth nerve palsies on the right side. The visual fields were full to confrontation in both eyes with intact visual acuity. MR imaging disclosed an enhancing soft tissue mass involving the clivus, pituitary fossa, cavernous sinus, and posterior nasal cavity (Figure 3) that surrounded both internal carotid arteries in the cavernous sinuses. An endonasal transsphenoidal approach to the sphenoid sinus was carried out to obtain a biopsy of the lesion. A histopathological diagnosis of metastatic melanoma was made, and evaluation was undertaken by the oncology team. (This patient was included in the cases described by McCutcheon et al. [13].)

### 3.4. Patient 4

**Figure 4:**
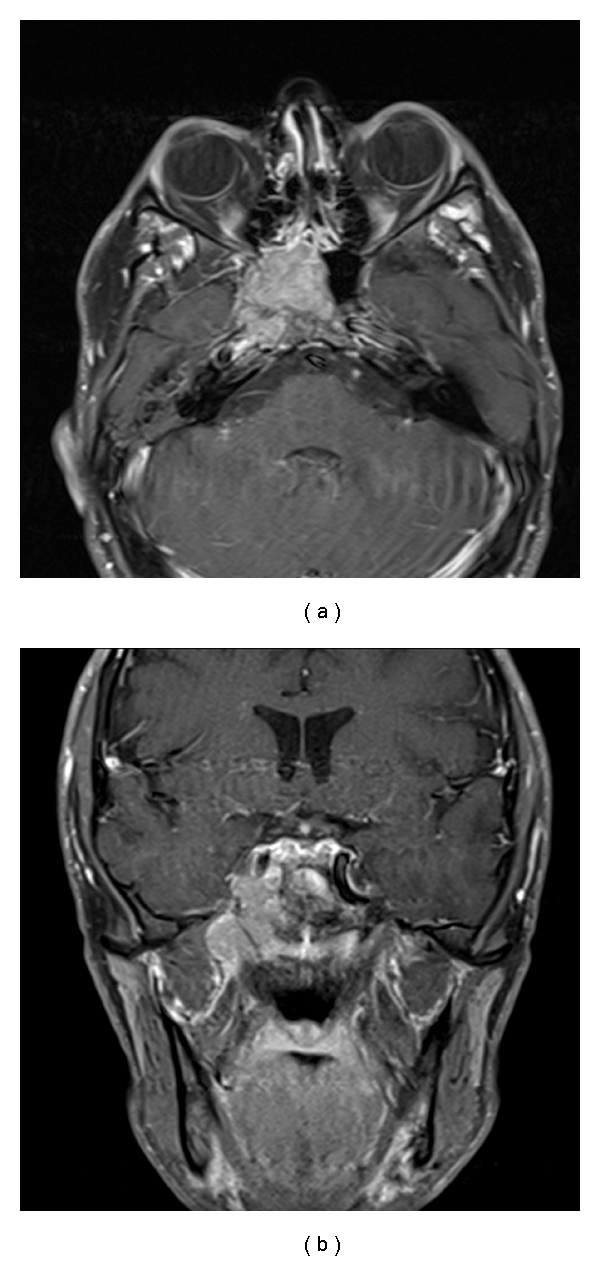
Axial and coronal T1-weighted, gadolinium-enhanced MR imaging revealing a homogenously enhancing lobulated lesion centered within the right sphenoid sinus with extension into the carotid canal, pterygopalatine fossa, and optic nerve canal.

A 21-year-old man with a remote history of osteosarcoma and newly diagnosed metastatic renal cell carcinoma had complaints of worsening vision and facial pain. Because there was a discrepancy in the pupillary size between his eyes, he underwent a computed tomography (CT) scan of the head, followed by craniofacial MR imaging, which revealed an enhancing mass in the right sphenoid sinus with adjacent extension. On neurological examination, he was noted to have complete hemifacial numbness and Horner's syndrome on the right side. MR imaging of the face demonstrated a homogeneously contrast-enhancing lesion centered within the right sphenoid sinus measuring 33 × 20 × 27 mm (Figure 4). The lesion extended into the carotid canal, pterygopalatine fossa, and optic nerve canal with destruction of the vidian canal and foramen rotundum on the right. No optic nerve involvement was recorded in the images. The patient was referred to the oncology service for radiation and possible chemotherapy.

### 3.5. Patient 5

**Figure 5:**
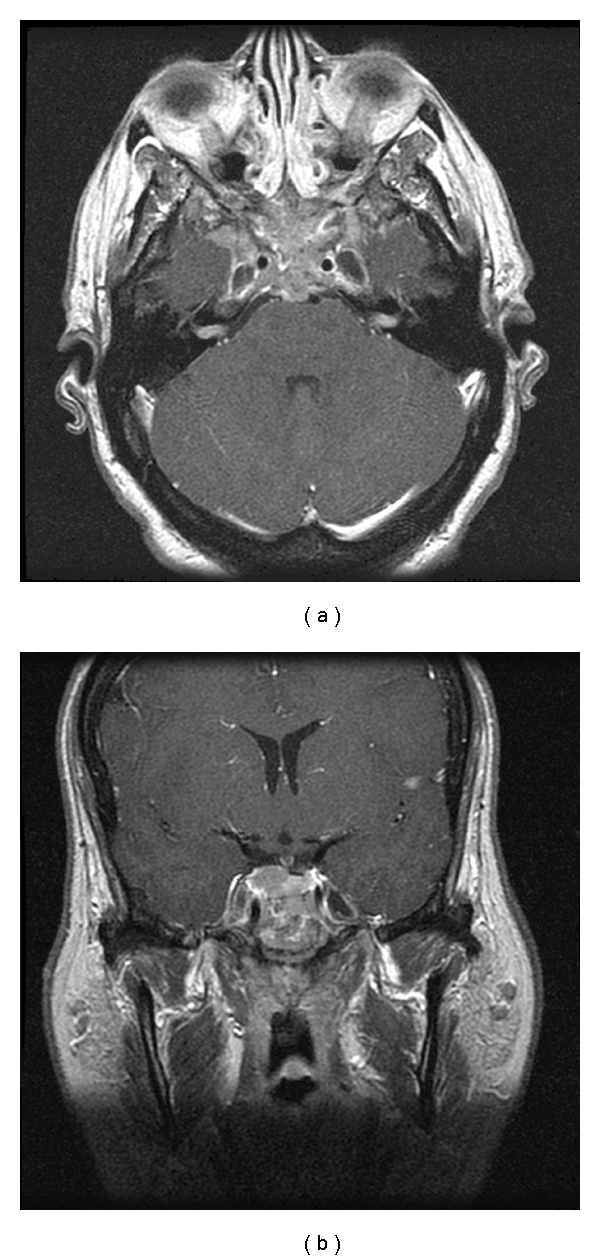
Axial and coronal T1-weighted MR imaging of the brain with gadolinium enhancement demonstrating a heterogeneously enhancing lobulated sellar and suprasellar lesion with extension into the right cavernous sinus and encasement of the right internal carotid artery.

A 42-year-old woman with several weeks of frontal headaches initially presumed to be secondary to a sinus infection underwent MR imaging after antibiotic medications failed to alleviate her symptoms. Her neurological examination was nonrevealing except for mild gait instability. Brain MR imaging demonstrated a lobulated, 25 × 20-mm sellar and suprasellar lesion with extension into the right cavernous sinus and encasement of the right internal carotid artery. This lesion was isointense on T1- and T2-weighted images and heterogeneously enhancing with gadolinium administration. A CT scan was consistent with a lytic lesion involving the central skull base extending laterally and posteriorly to involve the bilateral medial sphenoid wings and the clivus, respectively. A transnasal approach was undertaken to obtain a biopsy of the lesion. Histopathologic evaluation was consistent with a diagnosis of diffuse large B-cell lymphoma (Figure 5).

### 3.6. Patient 6

A 53-year-old woman presented with a known diagnosis of breast cancer and a one-year history of worsening left eye vision with a more precipitous decline in the last month. MR imaging of the brain demonstrated a skull base lesion involving the left sphenoid bone, the anterior clinoidal process, and the cavernous sinus. On neurological assessment, the patient was noted to have an acutely diminished visual acuity in her left eye to a level of finger counting with left temporal visual field cut. MR imaging (not shown) showed a homogenously contrast-enhancing lesion of the skull base involving the greater sphenoid wing and the anterior clinoidal process with encasement of the optic nerve on the left. A left frontotemporal craniotomy was performed for biopsy of the lesion and to decompress the optic nerve and the cavernous sinus with a subtotal resection. Histopathological analysis confirmed the diagnosis of metastatic breast carcinoma. The patient did not experience improvement in her vision postoperatively. She underwent fractionated radiotherapy to the involved area. One year after surgery, her neurological findings were unchanged, and her systemic disease was under control. 

## 4. Discussion

### 4.1. Tumor Sites of Origin

Neoplasms originating from a multitude of sites have been reported to metastasize to the SPS region. Breast and lung cancer account for approximately two-thirds of SPS metastases, being the most common sources in women and men, respectively, [11, 18–21]. Histological examinations of the tissue samples obtained during palliative hypophysectomy performed in patients with end-stage breast cancer and from autopsy series have documented metastasis to the SPS region in 6% to 29% of cases [3, 22–25]. Breast cancer metastasis comprised 33% of our cases presented in this study. One hypothesis put forth to explain this prevalence is that the prolactin-rich environment of the pituitary enhances the proliferation of breast tumor cells [10]. After carcinoma of breast and lung, lymphoma and prostate cancer have been reported to be the most common sources of metastasis to SPS region [6]. Liver, renal cell, colon, and thyroid cancers and melanoma are rare sources of distant metastases to this region. The relatively rarer occurrence of our other cases, prostate, melanoma, renal cell, and lymphoma, is consistent with the literature. Most cases are found in the sixth or the seventh decade of life as a part of a generalized metastatic spread, commonly associated with multiple, particularly osseous metastases [18, 20]; however, metastases can occur in young patients. The age of presentation ranged from 21 to 82 years in our small series, with an average age of 60 years. Very occasionally, these lesions are the first manifestation of an occult cancer or the only site of metastasis [14, 20]. Thus, in a patient without any prior history of cancer, an SPS lesion cannot be assumed to be an adenoma, just as in a patient with a known primary cancer, it is not always metastatic. Clinically, metastasis is generally suspected in patients with rapid onset and progressive symptoms, irrespective of a history of malignancy. 

### 4.2. Pathogenesis of Metastasis

The possible metastatic pathways to the pituitary and parasellar region include direct blood-borne metastasis to the posterior pituitary lobe, pituitary stalk, clivus, dorsum sellae, or cavernous sinus or leptomeningeal spread with involvement of the pituitary capsule [10, 26, 27]. There has been some controversy regarding the most common location of metastasis within the pituitary gland. Authors of early series have reported that the majority of pituitary metastasis occurs in the posterior pituitary, but some dispute this claim. Teears and Silverman [4] reported that 57% of the lesions localized to the posterior pituitary alone, 13% to the anterior pituitary alone, 12% to both lobes, and the remaining 18% to the capsule or stalk. They hypothesized that the posterior pituitary, by receiving direct arterial supply, is more likely to develop metastases than the adenohypophysis, which receives its blood supply from the hypophyseal portal system. The posterior lobe has a larger area of contact with the adjacent dura, which may be another contributing factor [4, 18]. Metastatic inoculation in the anterior lobe is usually the result of contiguous spread from the posterior lobe [28]. 

### 4.3. Clinical Presentation

Clinical symptomatology varies depending on the location of metastatic involvement. Cranial nerve palsies are the most frequent symptoms in cases of cavernous sinus metastases. These may be isolated, such as diplopia or ptosis, with the third (oculomotor) and the sixth (abducens) nerves being the most commonly involved, followed by the fourth (trochlear) nerve [14, 29–31], or they may appear in a constellation of symptoms characterized by unilateral, rapidly progressive ophthalmoplegia with retroorbital pain. This latter presentation is the usual presentation of the cavernous sinus syndrome, also known as parasellar syndrome [32]. If the branches of the trigeminal nerve are affected, alteration in the facial sensation, facial pain, or dysesthesia occurs [33]. Headache has been reported as a rather common symptom, with an incidence as high as 70% [14, 34, 35]; however, the majority of pituitary metastases are clinically silent. In the autopsy study by Teears and Silverman [4], only 7% of the pituitary metastases were symptomatic. These metastases are often seen in patients with terminal malignancy who present with malaise, generalized pain, central nervous system involvement, or treatment-associated symptoms, although symptoms of pituitary insufficiency may be masked. Several studies have indicated that diabetes insipidus (DI) was the most common symptom [10, 20, 24, 36, 37]. In the series of McCormick et al. [2], DI developed in 70% of patients; however, if the anterior pituitary function is compromised, DI may be concealed by reduced mineralocorticoid function [10]. Once the corticosteroid treatment is instituted, DI becomes clinically evident. In some recent series, DI has not been reported, which is likely because modern imaging techniques are able to detect abnormalities earlier than the timeframe required for DI development [35]. Other rarer hormonal findings may be hypothyroidism and hypoadrenalism, hypogonadism, or overproduction of adrenocorticotropic hormone (ACTH), growth hormone (GH), or prolactin [10, 38–40]. 

Because of their invasiveness, pituitary metastatic lesions have a high potential to cause visual deficits from suprasellar extension, with an incidence as high as 50% reported by Branch Jr. and Laws Jr. [14] and others [10, 34, 41]. In both the series by Chiang et al. [18] and that of Sioutos et al. [20], bitemporal hemianopsia was the most common type of visual impairment. Cranial nerve palsies involving the third and the sixth nerves and facial pain with numbness (trigeminal origin) were the most common presentation (33%) in our cases with cavernous sinus involvement. Retro-orbital pain, vision compromise, gait instability, Horner's syndrome, and hearing loss were infrequent and associated with petroclival and sphenoorbital extension of the lesions. All these symptoms were experienced with a relatively rapid onset from a few weeks to a few months that suggested the aggressive character of the lesions.

Symptoms strongly suggesting metastasis in the parasellar or sellar space include painful ophthalmoplegia in association with the sudden onset of DI [10, 26, 34, 42]. The pain may be retro-orbital or may be due to trigeminal dysfunction [43–45]. In our series, 5 of the 6 (83%) patients had a previous history of malignant disease. One of the patients that had prior history developed a malignancy (melanoma) other than the original one. Only one patient without a prior history of malignancy first presented with lymphoma metastasis to the SPS region.

### 4.4. Imaging

Because pituitary adenomas also present with invasion of the sellar floor, cavernous sinus, or clivus, no specific neuroimaging criteria to define metastatic lesions in SPS region have been reported. The diagnostic imaging tools for SPS metastasis mainly include high-resolution CT and MR imaging. Although CT is superior to MR imaging in detecting the bone involvement, the latter is preferable to determine the relationship of the lesion to the surrounding neurovascular structures [46]. Although nonspecific, the characteristics of these lesions on MR imaging are an iso- or hypointense mass on T1-weighted imaging with a usually hyperintense signal on T2-weighted imaging, and homogeneously enhancing mass in images obtained after the administration of contrast agent [47]. Invasion of the cavernous sinus, sclerotic changes around the sella turcica and clivus, isointense signal on both T1- and T2-weighted imaging, and loss of high-intensity signal in the posterior pituitary have been reported to be helpful in differentiating metastatic lesions from benign ones [15, 30, 48]. Morita et al. [10] and Komninos et al. [40] found that thickening or enhancement of the infundibulum was the most characteristic CT or MR imaging feature [15]. Schubiger and Halter [37] reported that the invasion of the infundibular recess by a suprasellar mass is suggestive of metastasis. Because of the rapid growth of metastatic lesions, a dumbbell-shaped intra- and suprasellar tumor with indentation at the diaphragm level is generally indicative for these cases [20, 37, 49]. The above-mentioned imaging characteristics are not specific for sellar or parasellar metastases. The radiodiagnostic findings that suggested a malignant/metastatic process in our cases were the involvement of multiple compartments in the anterior, middle, posterior cranial fossae, extension to the infratemporal and pterygopalatine fossae, sphenoid sinus, and nasal cavity with bony destruction in the cranial base and asymmetric or bilateral invasion into the cavernous sinus.

### 4.5. Clinical Management

The management of SPS metastases is multimodal, including surgical resection, radiation therapy, and chemotherapy [50]. Treatment is mainly palliative and depends on the symptoms and the extent of systemic disease [10, 51]. Because of the invasiveness and the high vascularity of the tumor, total surgical resection is generally not undertaken [1, 20]. Therefore, surgical treatment should aim for symptomatic relief and the preservation of visual function, even in patients with widespread primary disease, and should be followed by local radiation treatment and/or chemotherapy [10, 51]. The body of evidence is inconclusive on the effect of the latter two modalities on survival [14, 52]. Morita et al. [10] and Branch Jr. and Laws Jr. [14] reported improvement in symptoms, especially in pain and visual field defects, with no difference in survival after complete resection compared with subtotal or partial resection [10, 14]. On the other hand, others have supported the concept of improvement in survival after surgical resection of the lesion [53–55]. Surgical exploration is also essential if tissue diagnosis is likely to affect therapy in patients with no known primary malignancy. Resection is most commonly done via transsphenoidal route, although subfrontal or pterional approaches are also options depending on the location and the extension of the lesion. Four out of six patients in our series underwent surgical biopsy through either transsphenoidal or transcranial route and then underwent subsequent radiation/chemotherapy. One patient who had visual compromise had subtotal resection for palliation followed by radiation/chemotherapy. One patient who had renal cell carcinoma was directly referred to radiation oncology for immediate radiation and subsequent chemotherapy.

Besides its role as an adjunct after surgery [14, 42, 56], radiosurgery or conventional radiation is recommended as the initial course of treatment in patients with systemic disease out of control, recurrence in the systemic disease with concomitant SPS metastasis, or medical comorbidities that put the patient at risk for a surgical intervention [2, 7, 18, 41, 57–62]. Conventional radiation therapy can achieve symptom relief as high as 78% [62]. Radiosurgery, which is considered less invasive than conventional radiation, has been reported to achieve good tumor control [63, 64]. In a series of 23 patients by Iwai et al. [17], the rates of tumor control and symptom improvement were 67% and 53%, respectively; however, radiosurgery to the SPS region is limited by its potential to cause radiation injury to the surrounding neurovascular structures such as optic apparatus, pituitary gland, or cranial nerves coursing in cavernous sinus. Doses reported in the literature for these structures range from 8 to 40 Gy [63, 65–68], and the optimal dose may be quite variable depending on the proximity of the lesion. Furthermore, the debate about whether the radiation should be directed to the SPS region alone or to the entire brain continues.

Chemotherapy is commonly used alone or along with radiation therapy mostly for palliation in the treatment of metastatic disease in SPS region [69]. Its value has not been adequately studied and reported in the literature. 

### 4.6. Prognosis

The prognosis of patients with metastases to the SPS region is grim in a majority of cases because of the aggressive character of the primary disease [58]. Even in patients with no other metastasis at the initial evaluation, the prognosis remains poor because of radiologically undetectable microscopic metastases; however, it has been suggested that the extent of systemic disease affects survival in these patients [14]. Patients with a single SPS region metastasis may have a better outcome [10, 20]. Median survival is less than 2 years independent of the management strategy [10, 14, 70]. The records on the long-term follow-up of the majority of our patients are lacking because the patients were not monitored for surgical outcome, or the follow-up periods were too short. The only patient that had palliative surgery for vision compromise was seen at one year after the surgery with stable neurological findings.

## 5. Conclusion

Sellar and parasellar metastatic lesions are relatively rare. Breast and lung have been reported to be the most common sources in both sexes. Suggestive symptoms include rapid onset of progressive ophthalmoplegia with retro-orbital or facial pain, visual impairment, and/or DI. Management varies depending on whether a primary source is identified, the symptomatology, the location and extent of the lesion, the stage of the primary disease, and the medical comorbidities. Subtotal or partial surgical resection is aimed mainly for symptom relief. A multimodal approach involving subtotal resection of the lesion followed by radiation and/or chemotherapy is widely accepted, especially in symptomatic patients whose primary disease is under control. Radiation with or without chemotherapy is generally recommended as first-line treatment in patients with advanced primary disease or in those with high-risk medical comorbidities. A biopsy usually precedes radiation therapy if the primary source of the metastasis is unknown. The prognosis for patients is generally poor, independent of the therapeutic modality, and the overall survival is less than two years.

## Figures and Tables

**Table 1:** Clinical, endocrine, and radiological features of 6 patients with metastatic tumors of the sellar/parasellar region.

Patient	Age	Sex	Presenting symptoms	Symptom duration	Primary disease	Metastatic lesion	Location	Management	Outcome
1	77	m	Retroorbital pain Left nerve III palsy	4 months	Prostate cancer	Prostate cancer	CS and sella extending to inferior orbital fissure and upper clivus	Transsphenoidal biopsy, radiotherapy, chemotherapy	Stable neurological findings at two months

2	82	f	Hearing loss Facial pain Numbness in cranial nerve V1, V2	Several weeks	Breast cancer	Breast cancer	Petrous apex extending to Meckel's cave, lateral CS, and IAC	Transcranial biopsy, radiotherapy, chemotherapy	N/A

3*	79	m	Cranial nerve III and VI palsies	Several weeks	Prostate cancer	Melanoma	Bilateral CS, sella, clivus, and posterior nasal cavity	Transsphenoidal biopsy	N/A

4	21	m	Horner's syndrome Facial pain	1 month	Osteosarcoma Renal cell cancer	Renal cell cancer	CS extending to sphenoid sinus, pterygopalatine fossa, and optic canal	Radiation, chemotherapy	N/A

5	42	f	Gait instability Headache	1 month	Unknown	Lymphoma	Sellar/suprasellar lesion with bilateral CS, medial sphenoid wing, and clivus involvement	Transsphenoidal biopsy, chemotherapy, possible radiotherapy	N/A

6	57	f	Decreased visual acuity Peripheral vision defect	1 month	Breast cancer	Breast cancer	Greater sphenoid wing extending to the anterior clinoidal process with the optic nerve encasement	Transcranial decompression of optic nerve and the CS, radiotherapy with possible chemotherapy	Stable neurological findings and primary disease at one year

CS: cavernous sinus, IAC: internal acoustic canal, N/A: not available

*Patient was previously presented in McCutcheon et al.
